# Catalases Are NAD(P)H-Dependent Tellurite Reductases

**DOI:** 10.1371/journal.pone.0000070

**Published:** 2006-12-20

**Authors:** Iván L. Calderón, Felipe A. Arenas, José Manuel Pérez, Derie E. Fuentes, Manuel A. Araya, Claudia P. Saavedra, Juan C. Tantaleán, Sergio E. Pichuantes, Philip A. Youderian, Claudio C. Vásquez

**Affiliations:** 1 Laboratorio de Microbiología Molecular, Facultad de Química y Biología, Universidad de Santiago de Chile Santiago, Chile; 2 Laboratorio de Microbiología Molecular y Biotecnología, Facultad de Ciencias de la Salud, Universidad Andrés Bello Santiago, Chile; 3 Laboratorio de Microbiología Industrial y Biotecnología, Facultad de Ciencias, Universidad San Luis Gonzaga de Ica Ica, Perú; 4 Blood Testing Division, Chiron Corporation Emeryville, California, United States of America; 5 Department of Biology, Texas A & M University, College Station Texas, United States of America; Baylor College of Medicine, United States of America

## Abstract

Reactive oxygen species damage intracellular targets and are implicated in cancer, genetic disease, mutagenesis, and aging. Catalases are among the key enzymatic defenses against one of the most physiologically abundant reactive oxygen species, hydrogen peroxide. The well-studied, heme-dependent catalases accelerate the rate of the dismutation of peroxide to molecular oxygen and water with near kinetic perfection. Many catalases also bind the cofactors NADPH and NADH tenaciously, but, surprisingly, NAD(P)H is not required for their dismutase activity. Although NAD(P)H protects bovine catalase against oxidative damage by its peroxide substrate, the catalytic role of the nicotinamide cofactor in the function of this enzyme has remained a biochemical mystery to date. Anions formed by heavy metal oxides are among the most highly reactive, natural oxidizing agents. Here, we show that a natural isolate of *Staphylococcus epidermidis* resistant to tellurite detoxifies this anion thanks to a novel activity of its catalase, and that a subset of both bacterial and mammalian catalases carry out the NAD(P)H-dependent reduction of soluble tellurite ion (TeO_3_^2−^) to the less toxic, insoluble metal, tellurium (Te°), *in vitro*. An *Escherichia coli* mutant defective in the KatG catalase/peroxidase is sensitive to tellurite, and expression of the *S. epidermidis* catalase gene in a heterologous *E. coli* host confers increased resistance to tellurite as well as to hydrogen peroxide *in vivo*, arguing that *S. epidermidis* catalase provides a physiological line of defense against both of these strong oxidizing agents. Kinetic studies reveal that bovine catalase reduces tellurite with a low Michaelis-Menten constant, a result suggesting that tellurite is among the natural substrates of this enzyme. The reduction of tellurite by bovine catalase occurs at the expense of producing the highly reactive superoxide radical.

## Introduction

Molecular oxygen accepts electrons easily to form the reduced derivatives superoxide radical and hydrogen peroxide, which damage living cells. These reactive oxygen species and their products can modify nucleotide bases, cleave the phosphate backbone of DNA, crosslink proteins and lipids by free-radical driven chain reactions, and damage the active sites of critical enzymes. Organisms that thrive within or tolerate an oxygen-rich environment mount two critical lines of enzymatic defense against these reactive oxygen species. Superoxide dismutase (EC 1.15.1.1) converts superoxide to molecular oxygen and hydrogen peroxide [1], whereas catalases (EC 1.11.1.6) (and catalases/peroxidases) convert hydrogen peroxide to oxygen and water [2].

The monofunctional heme-containing catalases have been the subject of biochemical study for more than 100 years [3], and bovine liver catalase is among the first enzymes to be crystallized [4]. It is also among the few enzymes that have attained a catalytic efficiency equal to that limited by the rate of diffusion of its substrate, hydrogen peroxide. The disproportionation of hydrogen peroxide by catalase is dependent on a heme cofactor with a bound iron atom, which is cycled between oxidation states [2], [5]. Many catalases also have been shown to be peroxidases, and can oxidize short-chain alcohols including ethanol and other substrates in a two-step reaction dependent on hydrogen peroxide [6]–[8].

In addition to their active-site heme groups, monofunctional heme-containing catalases bind a second cofactor, NAD(P)H, which, surprisingly, is not required for its peroxide dismutase activity. This cofactor is bound so tightly by bovine liver catalase that it is not lost upon purification [9]. The X-ray analysis of bovine catalase shows that NADPH is located in a binding pocket with a novel architecture near the surface of the catalase tetramer, and makes no direct interaction with the more buried heme group of the enzyme. In the crystal structure, a water molecule is found adjacent to the reactive C4-carbon of the nicotinamide ring of NADPH, and fills a binding site for an unknown potential substrate, suggesting that NADPH is located at a second active site of the enzyme [10].

The role of NAD(P)H as a cofactor in the function of bovine liver catalase presents a biochemical mystery. Clearly, NADPH serves to protect this catalase against oxidative damage, presumably by tunneling electrons to the active-site heme group to regenerate its active ferricatalase form [11], [12]. Recently, it has been shown that the NADPH cofactor bound by catalases is reactive. In the case of eubacterial heme-containing catalase/peroxidases, the NADPH cofactor can be oxidized by molecular oxygen at high pH, and mediate both the hydrazinolysis of isoniazid and the synthesis of isonicotinyl-NAD to activate this synthetic anti-tuberculosis antibiotic [13]. These results explain why mutants of *M. tuberculosis* resistant to this nicotinamide derivative are found to carry mutations in the *katG* gene, encoding its catalase [14], [15]. However, isoniazid is not a natural antibiotic, and the natural substrates (electron acceptors) for a second, NAD(P)H-dependent, oxidoreductase activity of catalase have remained unknown to date. The oxidized NADP+ form of the bound cofactor of bovine catalase also participates in a redox reaction with free NADPH, which acts as both a proton an electron donor to reduce the NADP+ bound by the enzyme, and regenerate bound NADPH. However, free NADPH is oxidized more rapidly than bound NADPH during the dismutation of peroxide, suggesting that free NADPH plays a greater protective role than bound NADPH against the inactivating oxidation of the heme group by peroxide, a result that deepens the mystery of why bovine catalase binds NADPH so tightly [11], [16]. In the present article, we show that the NAD(P)H cofactor bound by monofunctional heme-containing catalases participates in yet another class of reactions, because we find that catalases can reduce the toxic heavy metal anion, tellurite, dependent on NAD(P)H.

The heavy metal tellurium falls below the essential elements sulfur and selenium in the periodic table of elements. One of the most oxidized forms of tellurium, tellurite (TeO_3_^2−^), is an anion that is toxic to both prokaryotes and eukaryotes, and is a very strong oxidizing agent. Among the prokaryotes, most Gram-negative bacteria are sensitive to tellurium salts, whereas some Gram-positive species are naturally resistant to tellurite [17]. Bacterial cells resistant to tellurite often turn black when grown in liquid or solid media amended with K_2_TeO_3_, a phenotype that is also displayed by sensitive bacteria growing in media supplemented with sub-lethal concentrations of tellurite [18]. These deposits are of an insoluble material localized to specific subcellular compartments [19], [20], which X-ray diffraction studies have shown to be the less toxic, free metal, tellurium (Te°) [21], [22].

To date, the precise biochemical mechanisms by which tellurite is reduced to tellurium in resistant bacteria have not been elucidated. Several oxidoreductases, including nitrate reductase and terminal oxidases of the bacterial respiratory chain [23], [24], can contribute to tellurite reduction. The nitrate reductase activities present in membrane fractions of the model eubacterium *E. coli* can mediate the reduction of tellurite. However, mutant strains with deficiencies of these enzymes retain a resistant phenotype under anaerobic conditions, arguing that the basal level of resistance is not due to this oxidoreductase [23]. In other bacterial species, the maximal rate of tellurite reduction is dependent on the presence of NAD(P)H or FADH_2_ cofactors [25]–[28], suggesting that tellurite reduction is coupled with the oxidation of these cofactors. In this article, we identify the primary oxidoreductases responsible for this activity in two bacteria, *S. epidermidis* and *E. coli*, as catalases.

## Results

### *Staphylococcus epidermidis* CH has an NADH-dependent tellurite reductase activity

**Figure 1:**
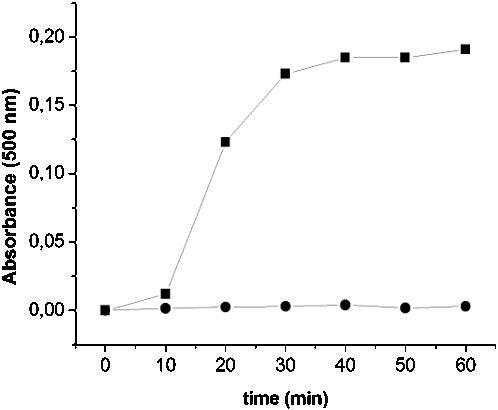
The tellurite reductase activity in crude extracts prepared from *S. epidermidis* CH is dependent on NADH as cofactor. The tellurite reductase activity present in crude extracts of *S. epidermidis* CH cells was followed over time as the increase in absorbance at 500 nm due to the conversion of tellurite to tellurium in the presence (▪) and absence (•) of NADH under standard assay conditions. The values shown represent the means of three independent determinations made with crude extracts containing 1.2 mg/ml total protein.

**Figure 2:**
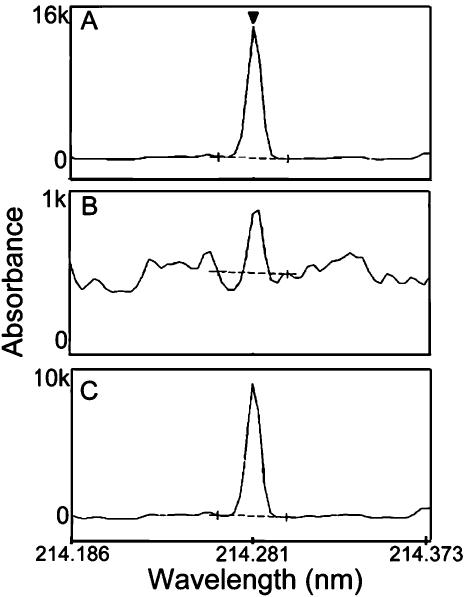
The product of tellurite reduction by catalase is elemental tellurium. Absorption spectra of products formed upon the reduction of K_2_TeO_3_ *in vitro* by **(A)** 2-mercaptoethanol, **(B)** a crude extract prepared from *S. epidermidis* CH cells prior to chromatographic enrichment, and **(C)** purified bovine liver catalase, were resolved by Induced Coupled Plasma-Optical Emission spectroscopy [30]. All products show an absorption maximum at 214.281 nm, the peak wavelength of the Te° standard (triangle in panel A). We note that the scale used to measure absorbance in the crude extract differs by a factor of ten from those used to measure absorbance in chemically prepared tellurium (A) and in the product of tellurite reduction by bovine liver catalase (C). This is because the crude extract includes a plethora of components with absorption maxima at or near this wavelength.

Recently, we described the isolation and characterization of a strain of the Gram-positive bacterium *Staphylococcus epidermidis* from the toxic effluent of a Chilean copper mine. *S. epidermidis* CH is resistant to high levels of tellurite, as well as to the salts of many other heavy metals. The minimal inhibitory concentration of tellurite for this microbe is 0.5 mM (150 µg/ml), 100-fold greater than that for the model eubacterium *E. coli* (1.5 µg/ml) [29]. To understand the biochemical basis for this resistance, we prepared cell-free extracts from *S. epidermidis* CH, and found that these extracts have an activity that catalyzes the reduction of K_2_TeO_3_, dependent on the presence of NADH (Fig. 1). This activity is sensitive to heat, proteases, sodium dodecyl sulfate (SDS), and guanidine hydrochloride, arguing that it is due to the presence of an enzyme. We confirmed that the product of tellurite reduction by the *S. epidermidis* CH extract is tellurium by using Induced Coupled Plasma-Optical Emission (ICP-OE) spectroscopy [30]. We compared the spectrum of the product produced by the extract with the spectra of metallic tellurium and potassium tellurite reduced chemically with 2-mercaptoethanol, as standards. The major peak observed when tellurite is reduced by a cell-free extract of *S. epidermidis* CH in the presence of NADH corresponds to that of metallic tellurium (Te°) (Fig. 2).

To purify the enzyme responsible for this NADH-dependent tellurite reductase activity, we fractionated the soluble proteins present in cell-free extracts of *S. epidermidis* CH using a series of chromatographic separations, and obtained a preparation enriched for this activity, as described in Materials and Methods. The enriched preparation was resolved by denaturing gel electrophoresis, and was found to contain two major proteins with apparent molecular masses of 75 and 60 kDa. The amino-terminal sequence of each of these two proteins was determined, and compared with those predicted to be of proteins encoded by the sequenced genomes of *S. epidermidis* strains ATCC12228 [31] and RP62A [32]. The amino-terminal sequence of the protein with higher molecular mass, QLLTSEQERKYPE, is most similar to that of oligoendopeptidase F (GenBank NP_764245), predicted to have a molecular mass of 69.7 kDa. That of the second protein, KQDGKLTXLFXAPV (X = an undetermined amino acid), is identical to that of catalase (GenBank NP_764571), predicted to have a molecular mass of 58.2 kDa. Because catalase has been shown to be an NAD(P)H oxidase [13], we reasoned that the NADH-dependent tellurite reductase activity in this fraction may be an activity of catalase.

### Catalases reduce tellurite *in vitro*

Heme-dependent monofunctional catalases differ in the tenacity with which they bind their NAD(P)H cofactor and in the substrate specificities of their accessory peroxidase activities. Whereas bovine catalase binds both NADPH and NADH very tightly, monofunctional bacterial catalases bind these cofactors less well. Both bovine catalase and the bifunctional *E. coli* KatG catalase/peroxidase have secondary peroxidase activities for a variety of substrates [6], [8], [13]. Although somewhat diverse in their biochemical properties, monofunctional heme-containing catalases have core primary sequences conserved among both prokaryotes and eukaryotes, suggesting that they have been acquired by eukaryotes upon lateral gene transfer from eukaryotes [33], [34]. Therefore, we made the simple assumption that, if the 60 kDa monofunctional heme-containing catalase present in the enriched fractions of a eubacterial cell-free extract mediates the reduction of tellurite, then bovine liver catalase also may be able to reduce K_2_TeO_3_ to Te° *in vitro*. We tested this hypothesis by assaying the activity of commercially available, purified bovine liver catalase. As shown in Fig. 2, bovine catalase also has an activity that reduces tellurite to tellurium *in vitro*.

**Figure 3:**
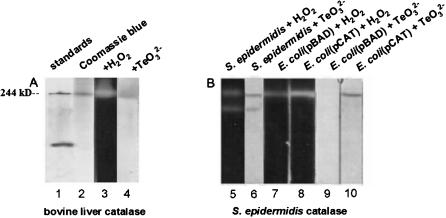
Catalases have tellurite reductase activity ***in situ.*** Assays for the H_2_O_2_ dismutase and TeO_3_^2−^ reductase activities of catalase *in situ* were carried out by resolving proteins on polyacrylamide gels, incubating gel strips in the presence of substrate, and developing gel strips to reveal the presence of specific products of each reaction (Woodbury et al. 1971; Gregory and Fridovich 1974). For dismutase assays, 25 µg of protein were loaded in each gel lane; 250 µg of protein was used for tellurite reductase assays. **(A)** The results in Fig. 3A show that bovine liver catalase (a tetramer of 255 kDa) migrates as a single band on native gels (lane 2) with an apparent molecular mass similar to that of a 244 kD standard (lane 1), and has both hydrogen peroxide dismutase (lane 3) and tellurite reductase (lane 4) activities. **(B)** The activities of catalase present in extracts made from *E. coli* Top10 cells carrying the plasmid vector pBAD display lower H_2_O_2_ dismutase and TeO_3_^2−^ reductase activities (lanes 7 and 9) than does an otherwise isogenic strain with plasmid pCAT, which expresses the *S. epidermidis* CH *katA* (catalase) gene (lanes 8 and 10); these activities most likely correspond to the predominant tetrameric form of catalase. In crude extracts of *S. epidermidis*, two different bands of each activity are observed (lanes 5 and 6).

The finding that purified bovine catalase reduces tellurite *in vitro* prompted us to develop an assay for this activity of catalase *in situ*, similar to a standard assay for its dismutase activity [35], [36]. The results in Fig. 3A show that when bovine catalase is resolved by electrophoresis on native polyacrylamide gels, it migrates as a single band with an apparent molecular mass similar to that of a 244 kDa molecular weight standard, representing the predominant, tetrameric form of the protein. As expected, the enzyme catalyzes the dismutation of hydrogen peroxide *in situ*, its tellurite reductase activity co-migrates with its dismutase activity.

To prove that the 60 kDa protein made by *S. epidermidis* CH is catalase, and can reduce tellurite *in vitro*, we amplified, then cloned, the *S. epidermidis* CH *katA* (catalase) gene into the *E. coli* plasmid expression_vector pBAD, as described in the Materials and Methods. This plasmid expresses cloned genes in response to the addition of the inducer, arabinose [37]. The recombinant plasmid (pCAT) was introduced into a wild-type strain of *E. coli*, and the expression of *katA* was induced by the addition of arabinose to transformed cells. Extracts prepared from exponentially growing cells were assayed for hydrogen peroxide dismutase and tellurite reductase activities *in situ*. Experiments were carried out in parallel with extracts prepared from exponential cultures of *S. epidermidis* CH and the otherwise isogenic *E. coli*(pBAD) strain (a negative control), which does not produce *S. epidermidis* catalase.

We find that the majority of hydrogen peroxide dismutase and tellurite reductase activities present in crude extracts prepared from *S. epidermidis* CH cells migrates as a band with an apparent molecular mass of 240 kDa when resolved by electrophoresis on native polyacrylamide gels (Fig. 3B).

*E. coli* cells also contain both a peroxide dismutase activity and a barely detectable tellurite reductase activity that migrate with an apparent molecular mass similar to that of the major activity found in *S. epidermidis* CH cell extracts. These activities correspond to the product of the *E. coli katG* (HPI catalase) gene, also a tetramer [38], because they are not observed in extracts prepared from otherwise isogenic, exponentially growing, *katG* mutant cells. Consistent with this result, a catalase-deficient, *katG* mutant strain of *E. coli* is more sensitive to tellurite than its otherwise isogenic, wild-type parent. Whereas the mutant strain has a MIC for tellurite of 0.75 µg/ml, the wild-type strain has a MIC of 1.5 µg/ml. Comparisons of the intensities of stained bands resulting from extracts prepared from *E. coli* cells with the pCAT plasmid expressing the *S. epidermidis katA* gene and otherwise isogenic cells carrying the control pBAD plasmid show that both the dismutase and tellurite reductase activities of catalase are expressed at more than-four fold higher levels from the pCAT plasmid. We note that about ten-fold more enzyme is necessary to detect the tellurite reductase activity of these catalases than to detect their dismutase activities *in situ*. This result is consistent with the observation that the NADH oxidase reaction mediated by bacterial catalases has a much slower turnover rate than does the dismutase reaction [13].

### Expression of the *S. epidermidis* catalase gene in *E. coli* results in increased resistance to tellurite

**Figure 4:**
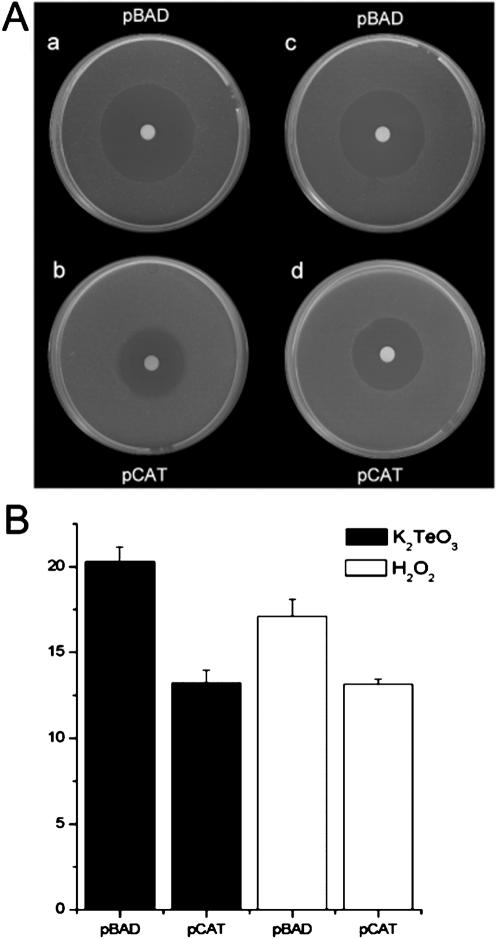
Figure 4. Expression of the *S. epidermidis katA* gene in *E. coli* confers increased resistance to both tellurite and hydrogen peroxide. **(A)** Aliquots of exponentially growing cultures of *E. coli* strain JWK3914 (*katG*) carrying plasmids pBAD (**a** and **c**) or pCAT (**b** and **d**) were spread onto the surface of plates with LB medium, 15.5 µl K_2_TeO_3_ (4 mM)(**a** and **b**) or hydrogen peroxide (3%) (**c** and **d**) were spotted onto the centers of the bacterial lawns, and cells were grown to reveal zones of inhibition (Fuentes et al. 2005). **(B)** The mean areas of growth inhibition zones (cm^2^) were determined from three independent experiments; thin bars represent the standard deviations.

To prove that the 60 kDa protein made by *S. epidermidis* CH is catalase, and is responsible for tellurite reduction *in vivo*, we tested whether expression of this gene in *E. coli* confers a higher level of resistance to tellurite. As shown in Fig. 4, the recombinant derivative of the *katG*-deficient *E. coli* strain JWK3914, which expresses the *katA* gene from plasmid vector pBAD, has a higher level of resistance to tellurite than does its otherwise isogenic parent with the pBAD vector alone. Indeed, the mutant *katG* strain of *E. coli* that expresses *S. epidermidis katA* has a slightly higher MIC of tellurite (1.75 µg/ml) than does the grandparental, wild-type strain of *E.coli* (1.5 µg/ml). This recombinant strain also displays a higher level of resistance to hydrogen peroxide than its otherwise isogenic parent. These results show that expression of the *S. epidermidis* catalase gene complements an *E. coli* deficiency, and support the idea that the product of the *S. epidermidis* CH *katA* gene is responsible for the high, natural resistance of this Gram-positive bacterium to tellurite *in vivo*.

### Bovine liver catalase evolves superoxide as a product of tellurite reduction, and has a low K_m_ for tellurite

The reaction scheme we propose for the reduction of tellurite by catalase is:
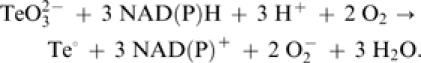



Thus, the reduction of tellurite by catalase requires molecular oxygen and produces superoxide as one of its products. This scheme is predicted to be favorable thermodynamically, involving three highly favorable half-reactions, the reduction of tellurite, the oxidation of NADH, and the formation of superoxide from oxygen. Consistent with the hypothesis that the reduction of tellurite produces superoxide, we have shown that an *E. coli sodA sodB* double mutant, deficient in superoxide dismutase, is hypersensitive to tellurite [39].

**Figure 5:**
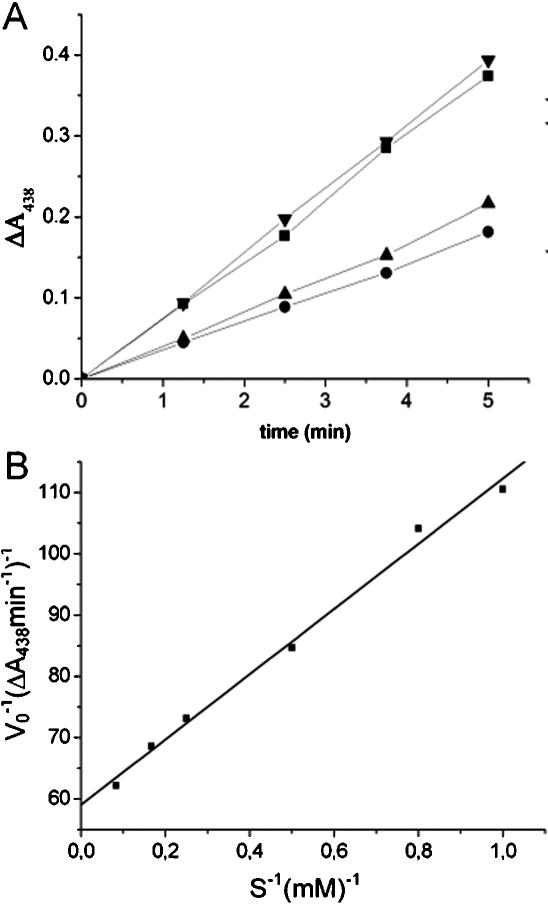
The reduction of tellurite by bovine liver catalase produces superoxide anion and obeys simple Michaelis-Menten kinetics. In these experiments, we measure the rate of evolution of superoxide radical as the product of the tellurite reductase reaction mediated by catalase, by the ability of superoxide to reduce the tetrazolium derivative WST-1. **(A)** The rates of change in absorbance at 438 nm in standard reactions with 5 mM K_2_TeO_3_ were measured. Reactions with tellurite (◂;complete) show a higher rate of WST-1 reduction than do reactions without substrate (•; - K_2_TeO_3_). Without substrate, WST-1 is reduced at a lower, background rate by NADPH. When superoxide dismutase is added to the reaction (▴; +SOD), only the lower rate of reduction is observed; in contrast, the addition of β-amylase (▪; +AMY) has little effect on the rate of reduction, demonstrating that the most abundant reductant of WST-1 in these assays is superoxide radical. **(B)** The reciprocals of the initial velocities of tellurite reduction, measured as the changes in absorbance at 438 nm min^−1^ due to the coupled reduction of WST-1, plotted versus the reciprocals of the concentrations of the substrate (S), potassium tellurite, (mM) for reactions carried out under standard conditions (see Materials and Methods for details).

To confirm that the reduction of tellurite by catalase results in the production of superoxide as a product, we determined the K_m_ of bovine liver catalase for tellurite, by assaying the rate of evolution of superoxide resulting from the reduction of tellurite by this enzyme. We detected the evolution of superoxide by virtue of its ability to react with the water-soluble derivative of tetrazolium, WST-1 [(2-(4-iodophenyl)-3-(4-nitrophenyl)-5-(2,4disulfophenyl)-2H-tetrazolium]. This reaction produces the reduced formazan form of the dye, which has a high extinction coefficient at 438 nm [40]. As shown in Fig. 5, superoxide is the reductant of WST-1 in this assay, because the addition of superoxide dismutase prevents the reduction of WST-1. Superoxide is produced at a rate dependent on the concentration of tellurite that follows simple Michaelis-Menten kinetics. Under these experimental conditions, catalase has an apparent K_m_ for tellurite of 0.9 mM. The K_m_ of catalase for tellurite is comparable to that for peroxide [2], arguing that it is one of the natural substrates of this enzyme.

## Discussion

A natural isolate of *S. epidermiditis* is highly resistant to tellurite. We have fractionated extracts from cells of this isolate, and find that an enriched preparation that catalyzes the NADH-dependent reduction of tellurite includes two major proteins. The amino-terminal sequence of one of these two proteins is that of catalase. Amplification and expression of the catalase gene from this isolate in *E. coli* results in increased tellurite reductase activity as measured *in situ*, and confers increased resistance to tellurite *in vivo*. Purified bovine liver catalase also has tellurite reductase activity, and produces metallic tellurite and superoxide as its products of tellurite turnover. Therefore, the NAD(P)H cofactor of catalase has a catalytic role both *in vitro* and *in vivo*, contesting the mystery of why bovine catalase binds this cofactor so tightly.

Our finding that *E. coli katG* mutants are more sensitive to tellurite than the wild type and can be complemented for this sensitivity by the expression of the *S. epidermiditis katA* gene solves yet another mystery: Why are mutants of *E. coli* deficient in nitrate reductase as resistant as their wild-type, parental strain to tellurite? The inducible enzyme, nitrate reductase (expressed at high levels only during anaerobic growth on nitrate as a terminal electron acceptor), does not appear to be the primary defense of *E. coli* against tellurite, as has been claimed. This prior claim is supported only by the results of assays of tellurite reductase activity *in situ* in the absence of NAD(P)H, conditions that preclude the detection of catalase as a tellurite reductase [23]. Rather, catalase, produced by *E. coli* during both aerobic and anaerobic growth [41], affords this primary line of defense together with superoxide dismutase [39].

Why is tellurite so toxic as to be reduced at the expense of generating the highly toxic superoxide radical? One intracellular target for tellurite appears to be thiol groups, because the exposure of aerobically growing *E. coli* to toxic levels of tellurite results in a decrease in the intracellular concentration of reduced thiol groups [42]. The oxidation of thiol groups by tellurite will generate superoxide as one of its products, and it has been proposed that tellurite may be toxic due to the production of this reactive oxygen species [43]. However, although we have found that both superoxide dismutase and catalase are necessary for resistance to tellurite *in vivo*, this explanation is unsatisfactory because the detoxification of tellurite by catalase also produces superoxide.

Rather, we favor the hypothesis that it is the other consequence of this reaction, the oxidation of proteins with active site thiols and iron-sulfur clusters, which accounts for the lethal effects of tellurite. The treatment of aerobically growing *E. coli* with tellurite results in the the loss of the transmembrane proton gradient and depletion of intracellular ATP without affecting the pools of glycolytic intermediates [44], suggesting that the primary targets for tellurite are critical carriers involved in respiratory electron transport. Several lines of evidence argue in support of this idea. When grown anaerobically without a terminal electron acceptor (employing mixed acid fermentation in rich medium), *E. coli* is ten-fold more resistant to tellurite [39]. We also have shown that over-expression of the *Geobacillus stearothermophilus* (formerly *Bacillus stearothermophilus*) *cysK* gene, encoding cysteine synthase (O-acetylserine sulfhydrylase; EC 4.2.99.8), in *E. coli* confers increased resistance to tellurite [45]. Similarly, an independent research team has shown that over-expression of the *Staphylococcus aureus cysM* (cysteine synthase) gene in *E. coli* also confers increased resistance to tellurite, and that inactivation of this gene in its natural Gram-positive host confers sensitivity to tellurite [46]. In addition, over-expression of the *G. stearothermophilus iscS* gene, predicted to encode a desulfurase (EC 2.8.1.7) involved in both the insertion of sulfur into iron-sulfur clusters and the repair of damaged iron-sulfur clusters, also mediates increased resistance to tellurite in *E. coli* [39]. The overproduction of both enzymes is predicted to result in an increase in the dissimilatory reduction of sulfate to sulfide by the *de novo* pathway for cysteine biosynthesis, and aid in the repair of damaged oxidoreductases.

A third mechanism that has been proposed to account for the toxicity of tellurite is that tellurium may replace sulfur in critical proteins with thiol groups or iron-sulfur clusters [47], leading to their inactivation or to the creation of telluroproteins with altered redox potentials. Selenocysteine is found at the active sites of oxidoreductases whose catalytic efficiencies and mechanisms can be altered by the replacement of cysteine [48]. Indeed, the substitution of tellurium for sulfur in the active site of subtilisin results in an enzyme with novel peroxidase activity [49]. It may be the case that, like selenite, tellurite is reduced to telluride on the same ATP carrier by the same enzymes that catalyze the assimilatory reduction of sulfite to sulfide, and that tellurite may compete with sulfite for incorporation into the active sites of oxidoreductases. However, this model cannot explain the result that inactivation of the *S. aureus* cysteine synthase gene, which should block the formation of telluroproteins by this route, results in an increased sensitivity to tellurite.

Catalase appears to be quite promiscuous, with two active sites that can participate in a variety of redox and condensation reactions. Tellurite is not the only heavy metal derivative that is a substrate of catalase. Both eukaryotic and prokaryotic catalases carry out the heme-dependent oxidation of metallic mercury, a reaction stimulated by hydrogen peroxide [50]–[53]. We suspect that catalase will have a wider range of substrates than those of which we are currently aware, and will play multiple roles in the defense against strong oxidizing agents encountered in nature, including a variety of heavy metal ions.

## Materials and Methods

### Fractionation of the tellurite-reducing activity from *S. epidermidis* CH

*S. epidermidis* CH cells were grown in LB medium containing erythromycin (50 µg/ml) at 37°C as described [29]. Cells were collected by centrifugation at 4,500× g for 10 min, washed, and suspended in PBG buffer (20 mM potassium phosphate pH 7.0, 1 mM 2-mercaptoethanol, 5% glycerol) at a ratio of 2 ml PBG buffer/g of cell paste. Subsequent purification steps were carried out at 4°C. After adding protease inhibitor (Sigma P-8465) to 0.5% v/v and lytic mixture [1 mg/ml egg-white lysozyme, 0.2 µg/ml of lysostaphin, 1 mM EDTA] to 10% v/v, cell suspensions were incubated at 37°C for 1 h. Glass beads were added, and mixtures were sonicated for 10 pulses of 30 s each at maximum intensity using a Ultrasonic Processor sonicator equipped with a microtip. Cell debris was discarded after centrifugation at 15,000× g for 15 min, and streptomycin sulfate was added to a final concentration of 2% w/v to precipitate nucleic acids. After 20 min incubation, nucleic acid complexes with streptomycin were pelleted by centrifugation at 10,000× g for 10 min, and the supernatants were retained. Subsequent chromatographic fractionations were carried out with resins from BioRad and Pharmacia, using PBG buffer.

Supernatants were loaded onto a Cibacron Blue column, connected in tandem to a carboxymethyl-Sepharose column. Tellurite reductase activity flowed through both resins, and was trapped on a DEAE-cellulose column. After washing this resin with five volumes of buffer, bound proteins were eluted with a linear 0–0.5 M NaCl gradient. Fractions with tellurite reductase activity, which eluted as a single peak at 0.2 M NaCl, were pooled, diluted to 0.05 M NaCl with PGB, and applied to a BioHTP column. Bound proteins were eluted with a linear gradient of 0–0.25 M potassium phosphate, and fractions with tellurite reductase activity were combined, diluted five-fold, and applied to a 0.5 ml BioHTP column for concentration. Proteins were eluted with 1 ml of PBG buffer supplemented to 0.3 M potassium phosphate, and dialyzed overnight at 4°C against PBG buffer containing 50% glycerol. Enzyme preparations were stored at −20°C. Extracts of *E. coli* cells were prepared by sonication and centrifugation, as described above for *S. epidermidis*. Proteins present in extracts and fractions were analyzed by polyacrylamide (12%) gel electrophoresis in the presence or absence of sodium dodecyl sulfate [35], [36], [54], and electroblotted to PVDF membranes as described [39].

### Standard assay for tellurite reduction and identification of the reduction product

Standard reaction mixes for assays of tellurite reductase activity contained 1 mM K_2_TeO_3_, 1 mM NADH, and fractions of cell extract or purified bovine enzyme in 150 µl PBG buffer. Tellurite reduction, measured at 37°C, was monitored by the increase in optical density at 500 nm (OD_500_) as described [28]. To identify the chemical nature of the black precipitate formed upon tellurite reduction, reactions were carried out for 2 h, then stopped by the addition of 250 µl 3 M NaCl. Mixes were centrifuged at 10,000× g for 10 min at 25°C, pellets were washed three times each with 0.1 M NaCl and distilled deionized water. The black pellets were dissolved in a solution of 10% bromine, and analyzed by Induced Coupled Plasma-Optical Emission (ICP-OE) spectroscopy [30], using an Optima 2000 DV Perkin-Elmer apparatus equipped with a Model 730 autosampler. Standards for this analysis were metallic tellurium, and the product obtained upon the chemical reduction of potassium tellurite with an excess of 2-mercaptoethanol; these were processed in parallel with the products of enzyme-catalyzed reactions. Bovine liver catalase (C-9322), superoxide dismutase (S-8160), and β-amylase (A-8781) used in these assays were from Sigma.

### Assays of catalase and tellurite reductase activities *in situ*

To assay for the presence of catalase and tellurite reductase activities *in situ*, samples were fractionated under non-denaturing conditions by electrophoresis in native polyacrylamide (12%) gels (40 mM Tris-glycine buffer, pH 8.3) at 4°C. To assay tellurite reductase activity, gel strips were incubated in 20 mM potassium phosphate buffer (pH 7) with 1 mM K_2_TeO_3_, 1 mM NADH, 1 mM 2-mercaptoethanol, and 5% glycerol at 37°C for 12 h. To assay catalase activity, gel strips were washed three times in distilled water for 15 min, submerged in a solution of 3 mM H_2_O_2_ for 25 min, rinsed briefly with distilled water, and developed in a solution of 1% w/v K_3_Fe(CN)_6_ and 1% w/v FeCl_3_ for 10–15 min until clear bands appeared [35], [36]. Reactions were stopped by washing gel strips with distilled water. Size standards in these assays included bovine liver catalase, bovine serum albumin, ovalbumin, lactate dehydrogenase, and egg-white lysozyme.

### Cloning and sequencing the *S. epidermidis* CH *katA* gene

The *S. epidermidis* CH *katA* coding region was amplified using wild-type genomic DNA as template with primers *kat1*, 5′ ATGTCAAAACAGGATGGAAAATTAAC and *kat*2, TTATTTAAAGTTTTCATATGTTTCGTCTTG. Primers were designed from the published sequence of the *S. epidermidis* strain ATCC12228 catalase gene, annotated as SE1016 in the genome sequence [27]. PCR reactions were carried out in a total volume of 100 µl using 2–5 ng of *S. epidermidis* CH DNA, 200 µM dNTPs, 1 µM of each primer, and 2.5 units of Taq polymerase (Perkin-Elmer). The amplified *katA* gene was purified and cloned into pBAD expression vector [37], using the pBAD TOPO TA Expression Kit (Invitrogen) to yield plasmid pCAT. Plasmid pCAT was introduced by electroporation into *E. coli* Top10, BW25113, and JWK3914 (*katG*) strains, the latter two of which were the generous gifts of Dr. H. Mori, Nara Institute of Science and Technology, Nara, Japan, otherwise isogenic strains generated as part of the international *E. coli* genome project.

To determine the sequence of the *S. epidermidis* CH *katA* gene, the amplified fragment was ligated to plasmid pET21 [55]. The 1,515 bp nucleotide sequence of the *S. epidermidis* CH *katA* gene has been deposited as GenBank acc. no. DQ301862, and differs by only 15 bp (less than 1%) from its homolog in the *S. epidermidis* 12228 genome sequence.

### MICs and growth inhibition zones determination

To determine the minimal inhibitory concentrations (MICs) of tellurite for each strain, cells were grown to overnight density in rich (LB) medium at 37°C, and diluted 100-fold into medium supplemented with various concentrations of K_2_TeO_3_ ranging from 0.0 to 2.0 µg/ml in 0.25 µg/ml increments. Growth was determined visually after incubation at 37°C for 48 h, and yielded the same, consistent positive or negative results in three independent trials. Zones of growth inhibition were determined as we have described previously [29].

### Kinetic analysis of the tellurite reductase activity of catalase

Standard reactions (1 ml) used to determine the initial velocities of tellurite reduction included 50 µl each of bovine liver catalase (7 mg/ml), NADPH (0.2 M), Tris-HCl (pH 8.0, 1 M), 20× WST-1 stock (Cell Technology, Inc.), and various concentrations of potassium tellurite. The increase in absorbance at 438 nm was monitored for 2 min; a linear response was observed for more than 5 min (see Fig. 5A). Control reactions without enzyme revealed a slow rate of spontaneous reduction of WST-1; this rate was subtracted as background, and was the same rate as observed in control reactions without substrate. Control reactions without the cofactor NADPH revealed nearly similar initial velocities of superoxide production, due to the fact that bovine catalase maintains four tightly bound molecules of reduced cofactor [9]. Control reactions with less oxygen, made by preparing reactants in the presence of nitrogen gas and overlaying mixtures of reactants transiently exposed to the atmosphere with mineral oil, yielded rates of reduction less than half of those observed without mineral oil.

To demonstrate that the reduction of tellurite by catalase produces superoxide as the reductant of WST-1, we carried out standard reactions supplemented to 5 mM K_2_TeO_3_ and to 200 µg/ml of either superoxide dismutase or β-amylase, as a negative control for the addition of a second enzyme to the assay. As a second negative control, we measured the rate of the reaction without K_2_TeO_3_, to determine the background rate of reduction of WST-1 by NADPH.
